# The rehabilitation enhancing aging through connected health (REACH) study: study protocol for a quasi-experimental clinical trial

**DOI:** 10.1186/s12877-017-0618-x

**Published:** 2017-09-20

**Authors:** Meng Ni, Lorna G. Brown, Danielle Lawler, Terry D. Ellis, Tamara Deangelis, Nancy K. Latham, Jennifer Perloff, Steve J. Atlas, Sanja Percac-Lima, Jonathan F. Bean

**Affiliations:** 1 Spaulding Rehabilitation Hospital, Boston, MA USA; 2 Department of PM&R, Harvard Medical School, Boston, MA USA; 3 College of Health and Rehabilitation Sciences, Boston University, Boston, MA USA; 4 Health and Disability Research Institute, Boston University School of Public Health, Boston, MA USA; 5 Heller School for Social Policy and Management, Brandeis University, Waltham, MA USA; 6 Division of General Internal Medicine, Massachusetts General Hospital, Boston, MA USA; 7 New England GRECC, VA Boston Healthcare System, Boston, MA USA; 8 Department of Exercise Science, Bloomsburg University of Pennsylvania, Bloomsburg, PA USA

**Keywords:** Physical therapy, Mobility, Geriatrics, Healthcare model

## Abstract

**Background:**

Mobility limitations among older adults increase the risk for disability and healthcare utilization. Rehabilitative care is identified as the most efficacious treatment for maintaining physical function. However, there is insufficient evidence identifying a healthcare model that targets prevention of mobility decline among older adults. The objective of this study is to evaluate the preliminary effectiveness of a physical therapy program, augmented with mobile tele-health technology, on mobility function and healthcare utilization among older adults.

**Methods:**

This is a quasi-experimental 12-month clinical trial conducted within a metropolitan-based healthcare system in the northeastern United States. It is in parallel with an existing longitudinal cohort study evaluating mobility decline among community-dwelling older adult primary care patients over one year. Seventy-five older adults (≥ 65–95 years) are being recruited using identical inclusion/exclusion criteria to the cohort study. Three aims will be evaluated: the effect of our program on 1) physical function, 2) healthcare utilization, and 3) healthcare costs. Changes in patient-reported function over 1 year in those receiving the intervention (aim 1) will be compared to propensity score matched controls (*N* = 150) from the cohort study. For aims 2 and 3, propensity scores, derived from logistic regression model that includes demographic and diagnostic information available through claims and enrollment information, will be used to match treatment and control patients in a ratio of 1:2 or 1:3 from a Medicare Claims Registry derived from the same geographic region. The intervention consists of a one-year physical therapy program that is divided between a combination of outpatient and home visits (6–10 total visits) and is augmented on a computerized tablet using of a commercially available application to deliver a progressive home-based exercise program emphasizing lower-extremity function and a walking program.

**Discussion:**

Incorporating mobile health into current healthcare models of rehabilitative care has the potential to decrease hospital visits and provide a longer duration of care. If the hypotheses are supported and demonstrate improved mobility and reduced healthcare utilization, this innovative care model would be applicable for optimizing the maintenance of functional independence among community-dwelling older adults.

**Trial registration:**

ClinicalTrial.gov Identifier: NCT02580409 (Date of registration October 14, 2015).

## Background

For adults aged 65 and over, a decline in mobility skills is a signal event, identifying higher risk for disability and increased healthcare utilization. Without detection and intervention, deterioration of mobility skills such as walking, climbing stairs or getting up from a chair can begin an inexorable downward spiral leading to dependency, morbidity, increased health care utilization and mortality [1, 2]. It is estimated that without establishment of new care paradigms specific to treating mobility limitations that these problems alone will add an estimated $42 billion to health care costs by 2040 [2]. Mobility limitations can be treated and there are opportunities for improving outcomes and access to quality focused care in both healthy and chronically ill older adults. Within a 2014 JAMA editorial [3], the importance of clinical programs targeting mobility was strongly emphasized as an important priority for an aging population. Rehabilitative care is indispensable in treating the functional consequences of diseases and conditions by enhancing physical function.

Currently, there is no drug therapy for mobility limitations and the most efficacious treatment is rehabilitative care. However, access to high quality rehabilitation services is a major gap for most healthcare systems. There are no established models that focus on treating and preventing mobility decline or that can be scaled to the varied populations of older adults residing in the US and other developed nations [1, 4]. An innovative approach is needed to optimize outcomes (physical and cognitive functions), minimize healthcare expenditures and facilitate retention of gains made in the skilled setting.

In the US, the federal program paying for care among adults 65 years and older, known as Medicare, is now mandating that primary care physicians perform an annual wellness visit that prioritizes preventative care strategies. Recognizing that screening of mobility skills is well suited for this sort of wellness visit, our study is designed to evaluate the benefits of a novel approach to rehabilitative care as a treatment within a preventative care paradigm. Primary care physicians do not typically prescribe rehabilitative care in this “ambulatory preventative care” context, and thus our program is not considered an example of standard practice. While a model of care targeting the secondary prevention of a decline in mobility skills among vulnerable older adults, is feasible within the traditional Medicare model of reimbursement [5], there are constraints on the duration and location (ambulatory versus home) of care. Thus, a course of physical therapy is often focused on limited, episodic care over a confined time duration (ie 4–8 weeks) that is segregated in either the home or an ambulatory care.

In a recently published clinical demonstration project evaluating a rehabilitative care program addressing mobility problems under Medicare reimbursement guidelines, robust improvements in mobility were observed even after accounting for health factors that might impede progress such as pain or cognitive impairment. This program targeted specific neuromuscular impairments identified as relevant to mobility and focused on other important elements such as principles of behavioral change [5–7]. However, limitations with program engagement and retention of patients were observed due to the frequency of outpatient visits required (average 10–12) and transportation limitations given that these patients manifested mobility limitations. Also, consistent with Medicare guidelines, this program provided treatment over an average of 6–8 weeks after which care was discontinued. Thus, long-term treatment and as a result benefits (i.e. over 6–12 months) were not evaluated. Interestingly, within participants from that clinical demonstration project, a consistent desire among participants was to receive similar treatment within a combination of both outpatient and home visits that were fewer in total number. However, participants wanted to be able to maintain contact with the PT, beyond 8–10 weeks, so that they could receive additional input to manage any problems that may arise.

In a separate clinical trial led by other members of this investigative team, longer-term mobility outcomes were targeted among community dwelling older adults recovering from hip fracture [8]. In that study, a video based exercise program was provided for 6 months to patients once they had completed their Medicare funded rehabilitative care. The intervention included approximately 2–3 training visits in the home with a skilled PT and then follow-up phone calls to promote compliance; while the control group underwent a cardiovascular nutrition education program. Interestingly, those in the intervention group demonstrated long term benefits with mobility beyond those achieved with normal care and the benefits were sustained 3 months after the intervention ended. This could be associated with the improved exercise self-efficacy at 9 months found in the intervention group.

Thus, we sought to develop a treatment program that built upon the strengths of this prior work. We desired a program that addressed the impairments linked to mobility decline, was conceptually grounded upon principles of behavior change and was able to provide longer term benefits in a fashion that met the needs of mobility limited patients. Therefore, we developed a program of care built around the use of a computerized tablet with a commercially available application designed to deliver home or community-based exercise. We conceptualized that this would enable us to deliver care within a relatively short number of sessions (approximately 8 visits) that were divided between outpatient and home visits, but delivered over an extended period of time (2–6 months). We chose a tablet-based application (App) that encompassed behavioral strategies such as goal setting, daily reminders, feedback, as well as a chat feature allowing for periodic communication with the treating physical therapist (PT). The PT focused on specific exercises as well as emphasizing engagement with exercise behaviors based upon their readiness for exercise and existing limitations and impairments. The combined provider settings (outpatient and home care) and App features allow PT to track exercise completion of the recommended exercise program in their home environment and offer modifications to maximize safety and benefit within the unique limitations of their physical space.

The uniqueness of this interventional protocol lies in the method of physical therapy delivery: 1) limited face to face treatment sessions spaced over a longer period of time, 2) remote monitoring for an extended period, 3) enhanced exercise performance with provision of videos/communications via an App, and 4) integration of targeting exercise and behavioral strategies. In Table 1, we describe some of the unique and innovative aspects of our rehabilitative care paradigm in contrast to the existing standard of care commonly prescribed for patients with mobility complaints.

**Table 1:** Comparison of rehabilitative care paradigm

Current traditional model	Proposed New REACH Model
Little to no planned contact with patients between skilled rehab visits	Regular contact via phone and the tablet via the App
Significant variability in the quality of visual aids/training for home exercise performance	High quality videos of the patients performing the assigned exercises with auditory feedback
Limited course of care over a relatively short period of time (episodic)	Care extended over a longer period of time with decreased frequency as patients assume more of their care independently-augmented by the App
Impairment focused interventional strategy targeting limited deficits	Function focused interventional strategy targeting comprehensive aspects of mobility
Behavioral change strategies are infrequently utilized in care for older adults	Incorporation of behavioral change strategies to encourage long term maintenance and adoption of exercise behaviors
Care typically delivered in one setting per episode of care	Mixture of home/outpatient visits to optimize safe, effective exercise performance and highlight environmental concerns
Limited ability to progress the exercise type and intensity as care episodes are of shorter duration	Extending the course of care over a longer period of time enabling program progression/modification/ as appropriate and able

## Methods

### Aims

We refer to our treatment approach as REACH (Rehabilitation Enhancing Aging Through Connected Health). REACH targets newly identified risk factors for mobility decline [7] and utilizes mobile health technology to deliver patient centered care more efficiently. Our project will evaluate three main objectives:

1. To evaluate the benefit of our mobility care program on physical function among 75 older adults at risk for mobility decline after one year of follow up in comparison to controls. We hypothesize that participants in our mobility care program will have significantly greater improvements in physical function after one year of follow up when compared to a matched control group of individuals not undergoing the REACH treatment.
2. To evaluate the impact of our mobility care program on health care utilization after one year of follow up by using Medicare claims data. We hypothesize that participants in our mobility care program will have significantly fewer hospitalizations and ED visits after one year of follow up when compared to matched controls over one year period of care.
3. To evaluate the impact of our mobility care program on health care costs after one year of follow up by using Medicare claims data. We hypothesize that after accounting for the estimated per patient costs of our intervention, participants in our mobility care program will have significantly lower healthcare costs after one year of follow up when compared to matched controls over one year period of care.

### Study design

This study is a quasi-experimental clinical trial (ClinicalTrial.gov Identifier: NCT02580409) conducted at the Spaulding Rehabilitation Hospital (SRH) and Boston University (BU). Participants are community-dwelling older adults aged 65 to 95 receiving primary care within clinics of the Massachusetts General Hospital (MGH). Collaborators from Brandeis University are leading the data analysis. This study is approved by the SRH Research Ethics Committee.

To most efficiently evaluate the effect of this novel program on mobility (aim 1), we aligned this intervention study with an existing longitudinal cohort study of older adult primary care patients at risk for mobility decline, known as the Boston Rehabilitative Impairment Study of the Elderly (Boston RISE). The Boston RISE methods are published elsewhere [9]. It evaluated changes in mobility annually over four years of follow-up among 430 primary care patients recruited from the Partners Healthcare System. For the current study, we are recruiting 75 additional primary care patients using the same inclusion and exclusion criteria. They will undergo a non-traditional mode of rehabilitative care and be compared to a matched control group derived from Boston RISE (control group 1). To evaluate the effect of the program on healthcare utilization and medical cost (aim 2 and 3), we will use data from fee-for-service Medicare beneficiaries residing in the Boston hospital referral region (HRR) (control group 2).

A propensity matching approach will be used to select participants for both control groups. Propensity scores will be modeled using logistic regression to predict the odds of participation using demographic and diagnostic information. Participants from the intervention group will be matched to similar participants from Boston RISE study and Medicare claims using propensity scores, age and sex. Those who have overlapping propensity scores (i.e. fall into the region of common support) will be candidates for matching. For matching, we will use a nearest neighbor approach with a 0.20 standard deviation caliper constraint. Since there will be many potential comparison candidates, we will consider a 2:1 or 3:1 match for each member of the treatment group. Although one-to-many matching introduces some bias in terms of the similarity between treatment and control participant, it leads to less variance for statistical testing of individual measures.

### Setting

Participants were identified through the through the MGH Primary Care Operations Improvement (PCOI) loyalty cohort (Protocol # 2004P002796) and direct identification by cooperating primary care providers. Eligibility was determined by a two-stage screening process, including using a Partners Healthcare patient database and telephone screening interviews. The initial onsite screening, baseline, 6-month, and follow-up assessment took place at the SRH and BU. Eligible participants completed baseline assessment within 2 weeks of the initial screening. Study staff contacted participants by phone every three months for a brief interview tracking falls, hospitalizations, Emergency Department visits, and rehabilitative care. Participants were scheduled to start their training sessions at SRH or BU, based upon geographic preference of the participant. Recruitment was initiated in August 2015 and completed in April 2016. Follow-up assessments are ongoing and will be completed in May 2017.

### Participants

Older adults living in the greater Boston area that were currently receiving primary care at MGH were recruited in this study. Potential subjects were sent a letter signed by their primary care physician (PCP) and the Principal Investigator (PI) describing the study and offering them the opportunity to state their disinterest in being contacted. Disinterested individuals could indicate their wishes by checking a box and returning a pre-paid postcard or contacting project staff directly by phone. If the potentially eligible primary care patient did not return the postcard or contact us within two weeks of receiving the letter, study staff contacted that individual, asking about their interest in participation. Interested individuals had their initial eligibility determined through completion of a 1-min telephone questionnaire designed explicitly for the identification of individuals at risk for mobility decline (those who respond that they have difficulty with or task modification in walking a ½ mile and/or climbing one flight of stairs) and through questions addressing exclusion criteria.

**Fig. 1:**
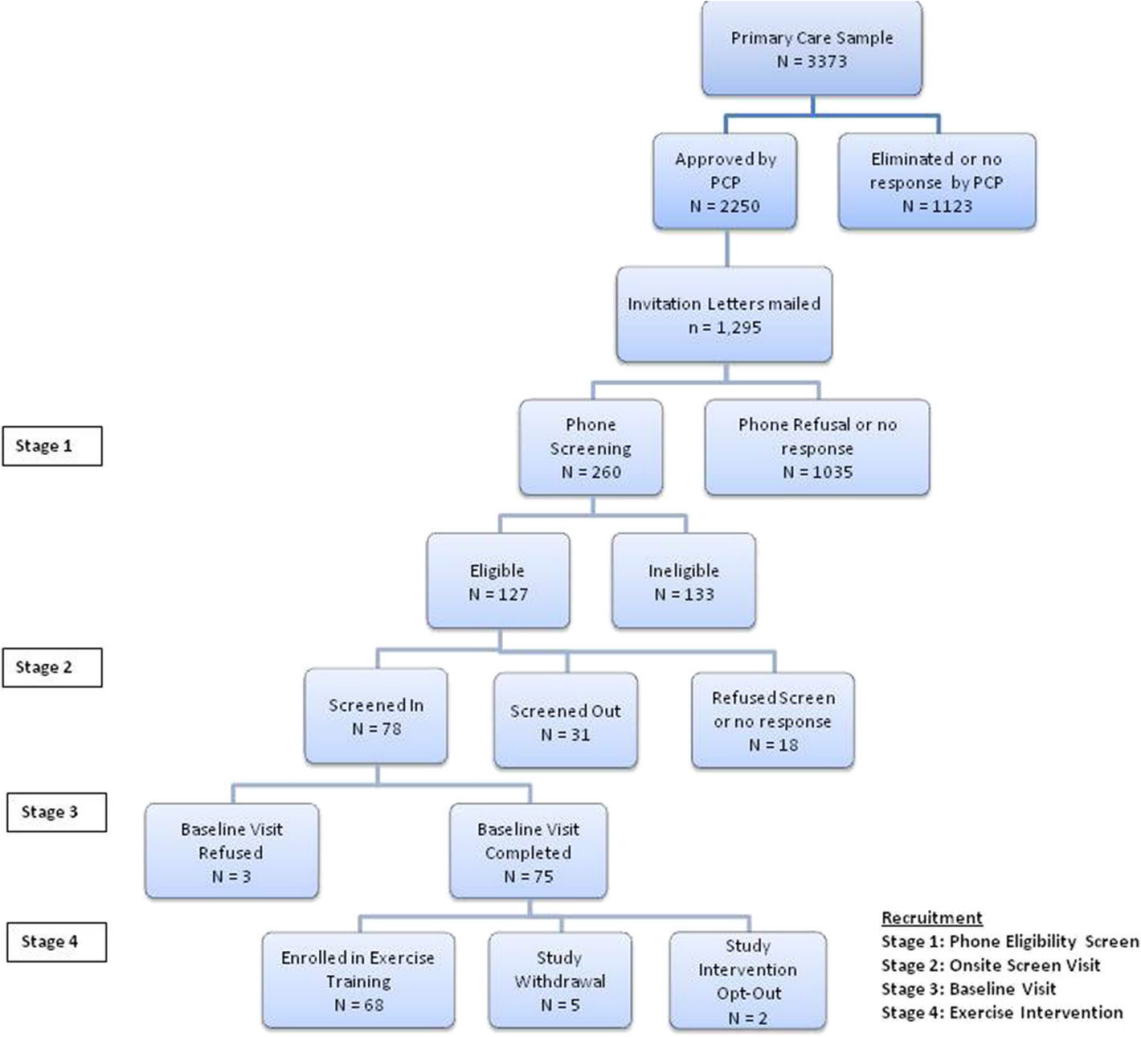
Participants screening and recruitment

Once the interested individual appeared eligible, they were promptly scheduled for initial onsite visit. After signing an informed consent form, participants underwent three tests, Mini Mental Status Exam [10], the Short Physical Performance Battery (SPPB) [11] and the 400 m walk test [12], to determine their final eligibility for study involvement. Once participants were determined to be eligible after those standardized tests, they were scheduled for a second visit for baseline evaluation. The procedure of participants screening and recruitment is presented in Fig. 1.

The inclusion criteria are:

- Age ≥ 65–95 years
- Able to understand and communicate in English
- Difficulty or task modification with walking ½ mile (6 blocks) or climbing one flight of stairs
- Ability to continuously walk 400 m in less than 15 min without stopping for more than a minute at a time, sitting, leaning, or the help of another person
- Lives in a zip code within 10 mile radius of Spaulding Cambridge Facility
- Baseline SPPB scores from 4 to 12 with ≤20% of SPPB scores in the 11–12 range

The exclusion criteria are:

- Presence of a terminal disease (e.g. receiving hospice services, metastatic cancer)
- Major surgery or Myocardial Infarction in the last 6 months
- Planned major surgery (e.g. joint replacement)
- Planned move from the Boston area within 1.5 years
- Mini-mental state exam (MMSE) score < 20
- Major medical problems interfering with safe and successful testing (examples may include: history hip replacement with recurrent dislocation, uncontrolled hypertension, use of supplemental oxygen)

### Outcome measures

In order to compare the participants’ characteristics with the control groups’, measures selected for this study were chosen based on those previously used in Boston RISE. The primary outcome measure is the change in patient reported function during the study period. It is evaluated by the Late Life Function and Disability Index (LLFDI), specifically utilizing the sub-domains of basic and advanced lower extremity function. The LLFDI is an interview-administered questionnaire that evaluates a broad range of functional limitations (inability to perform discrete physical tasks), in line with established conceptual models [13, 14]. The LLFDI-Function Category includes 32 physical tasks on a typical day without the help of someone else and without the use of assistive devices and participants were asked to report their current degree of difficulty in performing. Response options include: none, a little, some, quite a lot, cannot do. The scale is comprised of an overall function domain and three subdomains (for full description see [15]): upper extremity function, basic lower extremity function (e.g. standing, stooping, walking inside the home), and advanced lower extremity function (e.g. walking several blocks, getting up from the floor). The LLFDI has been shown to be valid and sensitive to change over two years [6, 15, 16], and its statistically relevant increments of change have been established by using minimal detectable change based on 90% confidence interval (MDC_90_) [6].

The secondary outcome measures are the SPPB and the 400-m walk test. The SPPB comprises three components: standing balance, usual walking speed, and a five times chair stand test. Scores from each component are added to create a score between 0 and 12, with higher scores indicating better performance. The SPPB and its components are valid and reliable test for predicting disability, nursing home admission, fall-related injury [17] and mortality [11, 18]. A one unit change in the SPPB has been characterized as a large clinically meaningful difference. [19, 20]. The 400-m walk is predictive of disability and mortality in older adults [21, 22]. Participants walk laps in a marked corridor with the goal to complete 400-m as quickly as possible [23]. Testing is terminated if participants take >15 min to complete the walk. The inability to complete the test in 15 min or less has been characterized as performance based measure of mobility related disability [3].

Additional assessments include cognitive and physical function tests, physiologic tests of neuromuscular attributes and participant self-report questionnaires. The collected variables and data collection time are presented in Table 2.

**Table 2:** Data collection time table

Assessment	Screen	Baseline	3 Month (Phone)	6 Month (in person & phone)	9 Month (Phone)	12 Month
Informed Consent						
Mini-mental state examination	X					X
Short Physical Performance Battery	X			X		X
Long Distance Corridor Walk (400-m walk test)	X					X
Demographic & Health History Questionnaire		X				
Technology Experience Survey						X
Physical Activity Item		X				
Comorbidity questionnaire		X				
Height/Weight & Vitals		X				X
*Cognitive Battery*		X				X
Hopkins Verbal Learning Test						
Trail Making						
Digit Symbol Substitution Test						
PHQ-9		X				X
History of Falls/Hospitalizations/ER/PT		X	X	X	X	X
Global measures of function & disability		X				X
Late Life Function and Disability Index		X		X		X
*Self-efficacy*		X				X
Activities Specific Balance Scale						
Barriers Specific Self-Efficacy Scale						
Brief Pain Inventory		X				X
McGill Pain Map		X				X
Computer attitude scale^a^		X				X
Grip Strength		X				X
Figure of 8		X				X
Trunk Extensor Endurance		X				X
Range of Motion		X		X		X
Leg Strength/Power		X				X
Stair Climb		X				X

Computer attitude scale^a^: evaluated after one week of starting the exercise program

Cognitive tests:

- Hopkins Verbal Learning Test [24]
- Trail Making (Parts A and B) [25]
- Digit Symbol Substitution Test [26]

Physical function tests:

- Figure 8 walk test [27]
- Stair climb power test [28]

Physiologic tests:

- Hand Grip Strength testing [29]
- Single leg press strength and speed testing [30]
- Knee/ankle range of motion [31]
- Trunk extensor muscle endurance test [32]

Self-report questionnaires:

- McGill Pain Map [33]
- Brief Pain Inventory [34]
- Katz Comorbidity Questionnaire [35]
- Depression (PHQ-9) [36]
- Activities Specific Balance Scale [37]
- Barriers Specific Self-Efficacy Scale [38]
- Computer Attitude Scale [39]

### Intervention and control group

#### Intervention group

Upon completion of the baseline assessments, participants were assigned to either of the two study locations for initiation of the exercise program and technology training with a licensed physical therapist. Participants can participate in an average of 6–10 in-person outpatient and home visits interspersed over a 9-month period. The visit frequency with an upper limit of visits of 12 sessions is tapered over the 9-month period with no in-person visits scheduled during months 10–12. Participants are videotaped performing the exercises using a tablet (iPad mini, Apple Inc., Cupertino, CA) and a corresponding web-based tablet App called Wellpepper (Wellpepper Inc., Seattle, WA). Auditory instructions from the physical therapist accompany each exercise video to optimize independent, high quality exercise performance. Each participant is provided their own tablet and trained in its use so they can appropriately access the App. This approach was used successfully by members of the research team within a separate study among community-dwelling older adults with Parkinson’s disease [40].

When the tablet is issued to the participant, their responsibilities regarding appropriate conduct and care with use of the tablet is reviewed and is designated by a signed agreement between the participant and study staff. Participants may keep (after all study data is cleared) or return the tablet to study staff after one year of participation in our study, or at early withdrawal. Home visits can be interwoven among the clinic visits to foster integration of the exercise program into daily routines with the support and guidance of the physical therapist. The frequency and timing of these visits are determined by the physical therapist based upon the individual needs of the participant. Remote monitoring through the App is tapered and continues as needed through the 9th month with independent performance of the program without PT’s initiated support for the last 3 months of the study duration (months 10–12). Participant adherence will be monitored through the App. Participants report whether they have performed an assigned exercise and can indicate the degree of difficulty and level of pain encountered. They can also send a message through the chat feature to report additional information they wish to share regarding performance of the exercise. The PT’s will respond to participant chat messages regularly and adjust the program as needed. If a participant goes 7 consecutive days without logging on to record exercise adherence, the PT is notified via email by the App. During the first nine months of the study, the PT will encourage adherence through messages in the chat, phone calls as needed and/or in-person visits. The optimal goal for performance of the exercise program 30 min each session, and engage in a walking program to tolerance. For those participants who struggle with development of a regular exercise habit, frequency expectations are lowered initially and progressed over time to achieve the goal of 5 times a week. Moreover, every time the subjects perform exercise, they also log whether or not they have experienced a fall in the last 24 h.

The exercises are based upon our prior research [8, 41, 42] and standard rehabilitative techniques advocated for older adults. They address attributes known to impact mobility such as leg strength, leg speed, trunk muscle endurance, limb flexibility, postural stability and walking function [5–7]. Exercises prioritize upright functional movements with progressive levels of difficulty and intensity with the goal of providing a safe, robust stimulus that is acceptable and has a high likelihood in enhancing function. A variety of exercises are included to guard against adaptation or boredom over the 12 month study. The exercises are not equipment based and lend themselves to safe, independent performance in the home environment. Each participant is assigned up to seven exercises at any given time. Exercises are progressed or modified in response to participant feedback. In addition, the participants are instructed in a progressive walking program. Exercises are prescribed and progress using a pre-determined set of exercises and progressions of each of these exercises (as described in Table 3).

**Table 3:** Progressive exercise program for leg strength, leg speed, trunk muscle endurance, limb flexibility, postural stability and walking function

Difficulty	Squat/sit to stand^a^	Step ups^a^	Plantar-flexors	Trunk Stabilization I	Trunk Stabilization II	Hip Girdle	Transitional to floor	Walking^b^
Level 1a	Elevated surface sit to stand	Single leg step up (with bilateral UE support)	Double leg heel raises with counter assist (forefoot elevated surface as needed)	Standing with back against the wall with heels, hips, shoulders and head touching the wall, keeping eyes level-arms down	Arm extended plank position using a counter for support, unilateral arm or leg extension	Standing chair/counter assist unilateral straight plane leg abduction	sitting - > half-kneeling - > sitting with chair/furniture assistance	Continuous 5–10 min
Level 1b	Sit to stand	Single leg step up (with single UE support)	Double leg heel raises with forearms resting on walls	Standing with back against the wall with heels, hips, shoulders and head touching the wall, keeping eyes level, add in alternating arm raises/double arm raises	Arm extended plank position using a counter for support, alternate arm/leg extension	Standing wall finger tip assist unilateral straight plane leg abduction	sitting - > quadraped - > sitting with chair/furniture assistance	Continuous 10–20 min
Level 2a	Touch and goes (Buttock barely comes to rest on the chair followed by a rapid stand)	Single leg step up without UE support	Double leg concentric raise/single leg eccentric lower		Quadraped alternate arm lifts	Standing no UE assist unilateral straight plane leg abduction	sitting - > quadraped - > supine - > sitting with chair/furniture assistance	Continuous 10–20 min with interval bursts
Level 2b	Chair hover (Butt not allowed to touch the chair (hover) followed by a rapid stand)	Single leg step up with opposite foot toe tap on the step	Single leg concentric/eccentric raises with counter assistance		Quadraped alternate arm/leg lifts	Wall side plank unilateral hip abduction	Standing - > half-kneeling - > standing no chair/furniture assistance; Reverse lunge with no chair/furniture assistance	continuous 20–30 min
Level 3a	air squats or wide leg squats	Single leg step up with opposite foot hover over the step	Single leg concentric/eccentric heel raises with wall assist		Prone alternating arm/leg lifts	Air squat with single leg abduction upon rising	Standing - > quadraped - > standing, no chair/furniture assistance	Continuous 30+ minutes
Level 3b	Tandem sit to stand or wall squat with hold	Single leg step up with opposite leg hip flexion	Increasing reps/excursion of the motion		Prone superman or standing founder	Single leg dead lift	Standing - > supine- > standing, no chair/furniture assistance	Continuous 30+ with interval bursts
Stretch/Low resistance option	Figure 4 stretch seated or supine	Unilateral standing hamstring stretch on stair	Unilateral calf stretch in standing lunge position		cat/cow stretch in quadraped	Single/Double Knee to chest in supine	Unilateral hip flexor stretch in high kneeling or standing	standing arms up/down with breathing

^a^Rapid concentric motion with slow/controlled eccentric motion

^b^Increasing walking speed, either continuously or during shorter burst throughout the walk

#### Control groups

The Boston RISE study utilized an identical recruitment strategy to what is employed for this study, but did not receive any formal treatment as this was an observational cohort study. For aim 1, approximately 150 matched controls will be selected from the Boston RISE study to serve as a control group. For aim 2 and 3, a group of approximately150 individuals’ Medicare claim data will be selected based on clinical similarity to participants in the physical therapy trial.

### Statistics and sample size

We sought to recruit 75 participants in the intervention group based on power calculations (power ≥ 0.8 at an effect size of 0.5) predicting a difference in change score of 6.31 units in the LLFDI advanced lower extremity function sub domain [6], while accounting for a dropout rate of 15% of participants over one year of follow-up.

This quasi-experimental study will use a difference-in-difference (D-in-D) approach to assess the impact of REACH compared to usual care. We will use strong matching algorithms to ensure comparability on observable characteristics between members of the treatment and two control groups. More specifically, for the first control group (Boston RISE), we will match participants on functional status, age and sex using a nearest neighbor approach. For the second control group (Medicare claims), we will use beneficiaries residing in the Boston hospital referral region. We will flag demonstration participants and then model the probability of being a member of the treatment group.

The basic model estimates the effect of treatment on the change in outcome between baseline and follow-up: *y*_*it*_ = *β*_0_+*β*_1_ *treatment*_*i*_ +*β*_*s*_ *post*_*it*_ + *β*_3_ *treament*_*i*_^∗^ *post*_*it*_ + *γcontrols*_*it*_ + *α*_*i*_ + *ε*_*it*_ where *y*_*it*_ is cost, *γcontrols*_*it*_ are patient socio-demographic characteristics and *treament*_*i*_ is a 0/1 variable indicating treatment status. *β*_3_ is the parameter of interest, capturing the joint effect of being in the treatment group at time 2. For the first study aim, this model can be extended to include repeated measures of functional status over time.

## Discussion

To our knowledge, REACH represents a unique approach to delivering physical therapy for community-dwelling older adults. The aim of this report is to describe the methods and rationale for assessing the preliminary effectiveness of the REACH intervention. If our hypotheses are correct, these findings will demonstrate the potential benefit not only in terms of physical functioning, but also from an economic perspective providing useful information for payers and accountable care organizations.

Home exercise integration is highlighted as the strengths of this novel healthcare model. According to the findings from another clinical demonstration program for older patients [5], there were barriers limiting program engagement, including transportation, weather, and time management. Therefore, the proposed healthcare model with an emphasis on home-based modes of preventive and rehabilitative care may be viable to address these individuals’ needs. Another highlighted feature of this program is use of a computer application that through the use of prompts and feedback helps reinforce and optimize exercise behavior [8]. Meanwhile, considering other potential influential factors such as high levels of comorbidity and poor exercise readiness, the physical activity sessions in our program are individualized and progressive, aiming to better address individuals’ needs and increase readiness for exercise [5]. A structured and moderate-intensity physical activity program provided over a long duration (up to 2.5 years) can prevent the onset of major mobility disability and favor improved recovery in individuals who lose mobility [3]. However, clinical models of care targeting mobility decline that are economically feasible within current Medicare funding structure has yet to be identified. This investigation will evaluate if the REACH intervention may be identified as a potential means of treating mobility problems within an economically feasible approach.

As demonstrated in Table 4, baseline SPPB scores indicate a mean score of 8.9, which is considered a moderate risk for functional decline. Also, the participants in our cohort have a mean of approximately four chronic medical conditions. This level of functional limitation and comorbidity is consistent with levels observed among community dwelling older adults residing in this region [43]. Our trial uses measures that are valid among older adults of varying health status. The staff has extensive experience conducting these measures safely among older adults with mobility problems. Many of the assessments for this study involve minimal risk to the participants. It increases the feasibility and applicability of this exercise program in clinical settings. The findings from our study will be distinctively applicable for translation into a multidisciplinary care program that includes both primary and rehabilitative care.

**Table 4:** Participants characteristics at the baseline

	Mean (SD) or N (%)	Range
Age	77.77 (6.07)	67–92.6
Gender		
Female	41 (54%)	
Male	35 (46%)	
Hispanic of Latino ethnicity	0	
Race		
White	63 (83%)	
Black	5 (6.5%)	
Other	8 (10.5%)	
BMI		
< 25	23 (30%)	
25.0–29.9	36 (47%)	
> =30	17 (23%)	
Number of chronic medical conditions	3.93 (1.91)	1–9
SPPB	8.92 (1.86)	4–12
400 m walk (min)	6.44 (1.82)	3.75–14.22
LLFDI – Overall Function	58.27 (7.41)	42.22–81.67
LLFDI – basic L/E function	68.63 (10.74)	48.52–100
LLFDI – Advanced L/E function	47.53 (11.64)	18.11–81.63

*SD* Standard deviation, *SPPB*, Short Physical Performance Battery, *LLFDI* Late Life Function and Disability Index, *L/E* Lower extremity

We acknowledge that our study findings may be difficult to generalize to older adults residing in other countries or other regions of the United States. However, if we are able to observe preliminary effectiveness, it will justify evaluation and study at a larger level across varied care settings targeting community dwelling older adults. Also, despite, this potential limitation, our study is strengthened by its grounding within a model of clinical care.

## Abbreviations

App: Application
Boston RISE: The Boston Rehabilitative Impairment Study of the Elderly
BU: Boston University
HRR: Hospital Referral Region
LLFDI: Late Life Function and Disability Index
MDC_90_: Minimal detectable change based on 90% confidence interval
MGH: Massachusetts General Hospital
MMSE: Mini-mental state exam
PCOI: Primary Care Operations Improvement
PCP: Primary care physician
PI: Principal investigator
PT: Physical Therapist
REACH: The Rehabilitation Enhancing Aging Through Connected Health
SPPB: Short Physical Performance Battery
SRH: Spaulding Rehabilitation Hospital
